# Impact of socioeconomics on recurrences and survival in non-metastasized colorectal cancer

**DOI:** 10.1038/s41416-025-03224-w

**Published:** 2025-10-04

**Authors:** Erik Osterman, Elisavet Syriopoulou, Anna Martling, Therese M.-L. Andersson, Caroline Nordenvall

**Affiliations:** 1 Department of Molecular Medicine and Surgery, Karolinska Institute, Solna, Sweden; 2 Department of Medical Epidemiology and Biostatistics, Karolinska Institute, Solna, Sweden; 3 Department of Pelvic Cancer, Colorectal Surgery Unit, Karolinska University Hospital, Solna, Sweden

**Keywords:** Colorectal cancer, Epidemiology, Outcomes research

## Abstract

**Background:**

Survival differences between socioeconomic groups in colorectal cancer have been studied for patients diagnosed in the 90s and 00s, but research on recent patients using individual measures of socioeconomic position is limited.

**Methods:**

CRCBaSe, a database of linked national registry data, was used to analyse stage I–III colorectal cancer patients diagnosed in Sweden between 2008 and 2021. The exposures of interest were income and education. Flexible parametric survival models were fitted and standardised survival probabilities and hazard ratios (HR) were calculated for cancer-specific survival, recurrence, and overall survival.

**Results:**

Analysis of 59,995 patients showed better 5-year standardised cancer-specific survival in the least deprived income group, 77.8% (95%CI 76.9–78.6) vs. 73.2% (95%CI 72.6–73.9) in the most deprived income group, HR 0.93 (95%CI 0.87–0.99). Time to recurrence was not statistically different between socioeconomic groups. Overall survival was better in the least deprived income group, with a 5-year standardised overall survival of 70.0% (95%CI 69.1–70.8) vs. 63.5% (95%CI 62.9–64.1) in the most deprived income group, HR 0.82 (95%CI 0.79–0.86).

**Conclusion:**

We found large disparities in cancer-specific and overall survival between the highest and most deprived income and education groups, despite improvements in care and the introduction of guidelines.

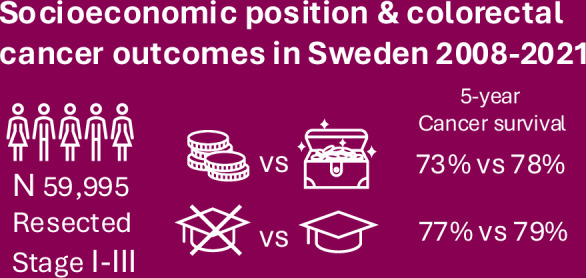

## Introduction

Is the prognosis after colorectal cancer (CRC) resection dependent upon socioeconomic position (SEP) in a modern material? Previous studies show a substantial variation in CRC survival by SEP, age and to some extent sex [1, 2]. Differences by SEP persist even in countries with universal healthcare access and irrespective of how deprivation is defined, with the most deprived individuals having the worst prognosis. Many studies on the subject have been performed before the introduction of national and international guidelines, and the establishment of national quality registries. This has also been the case in Sweden where regional guidelines existed in the late 00s and national guidelines were introduced in 2016 [3–9]. The quality of care has improved during the last decades and evaluations based on earlier data are probably not representative of the current practices [10, 11]. Today, nearly all elective colorectal cancer patients are discussed at preoperative multidisciplinary team conferences (MDT), yet differences in treatment remain [12, 13].

The most deprived are more likely to have emergency surgery [3, 12] and differences can be seen across SEP for oncological, surgical treatment [12, 14, 15], and outcomes [16]. In data from England, relative survival differences of 10% between socioeconomic groups have been observed [17]. Some of this is explained by differences in prognostic factors [18], but it is unlikely to be entirely through such factors [19]. Most previous studies have focused on overall survival, which captures both the effect of recurrences and mortality from other causes, without distinguishing these two [20]. Cancer-specific survival is useful for assessing the risks and benefits of treatment while recurrence rates or time to recurrence measures if the cancer treatment was successful and highlights treatment and stage differences between groups.

This study aimed to investigate differences in time to recurrence, cancer-specific survival, and overall survival for stage I–III CRC in Sweden across socioeconomic groups. The hypothesis was that the most deprived have more recurrences and worse survival outcomes despite improvements in the quality of care.

## Materials and methods

The study was approved by the Regional Board of the Ethical Committee in Stockholm (DNR: 2014/71-31, 2018/328-32) and by the Swedish Ethical Review Authority (DNR: 2021-00342, 2023-03305-02).

### Patient data

Patient data originated from the Colorectal Cancer Database (CRCBaSe), a register-linkage of the Swedish Colorectal Cancer Registry (SCRCR) and national registries at the National Board of Welfare and Statistics Sweden described in more detail elsewhere [21, 22]. Registry data were linked using the Swedish personal identification number, the unique number issued to everyone living in Sweden.

All adults (≥18 years old) with a resected stage I–III CRC in CRCBaSe with a first-time diagnosis between 2008 and 2021 were included in the study.

### Exposures

SEP was defined based on two indicators, the individual part of disposable household income and the highest education achieved, with income being the main exposure of interest. These variables are collected by Statistics Sweden yearly from other government agencies. The average individual part of disposable household income 2 years before diagnosis was split into sex and age-specific quartiles to allocate patients in groups of high and low socioeconomic positions (cut-points in Supplementary Table 1). The quartiles were created separately for patients above and below 65 years of age of the same sex with income quartile 1 (Q1) being the most deprived, and income quartile 4 (Q4) being the least deprived. The highest level of education attained at diagnosis was categorised as <9 years, 9–12 years and >12 years, with <9 years corresponding to the least educated (and most deprived) and >12 years to the most educated (least deprived).

### Outcomes

The outcomes of interest were time to recurrence (TTR) from surgery and cancer-specific survival (CSS) from diagnosis. In addition, overall survival (OS) was investigated [20]. Follow-up for TTR was set to 6.5 years after diagnosis since the mandatory reporting to the registry stops at 5 years (with some delay). Cases with events or follow-up beyond this period were censored at 6.5 years.

### Other covariates

Civil status at diagnosis was classified into two categories (living alone or not) reported by Statistics Sweden based on registered marriages, divorces, partnerships and cohabitation information from the census. For those with missing information on civil status, individual and household income data was used to infer their civil status. Specifically, patients with a ratio of individual income to weighted household income smaller than 0.9 or larger than 1.1 were reclassified as living with someone even if they were unmarried or not in a registered partnership.

Comorbidities were measured using the Charlson Comorbidity Index (CCI) [23] calculated before cancer diagnosis from the National Patient Registry, containing all diagnoses from in- and outpatient care since the 1980s [24]. In addition, the American Society of Anesthesiologists physical status classification (ASA) reported in the SCRCR and based on the preoperative anaesthesia evaluation was included [25]. Compared to the Charlson comorbidity index, the ASA classification captures functional limitations instead of relying solely on previous diagnoses.

Age at diagnosis, sex, year of diagnosis, tumour location and pT and pN stage was obtained from the SCRCR and included as confounders in the models.

### Statistical methods

No power analysis was performed since the study design called for using all available cases in the CRCBaSe. Complete case analysis was used since there were few cases with missing data for the exposures, covariates and outcomes. Demographics were obtained by income and education and presented as the number of patients in each group and the proportion in each group. Kaplan–Meier graphs for OS by income and education groups including the number at risk throughout follow-up were drawn, corresponding to unadjusted estimates.

Data were analysed using flexible parametric survival models (FPM) with 4 knots for the baseline hazard shape to adjust for confounding [26, 27]. Knots were placed at equally distributed quantiles of the log of the event times. Direct acyclic graphs were used to assess potential confounders (Supplementary Fig. 1). Separate FPM were fitted for each of the exposures of interest (income and education) and these were adjusted for civil status (partner/no), year of diagnosis (year), sex (male/female), age (continuous with restricted cubic splines to allow a non-linear effect of age with 3 knots), Charlson Comorbidity Index (CCI, continuous) [23], American Society of Anesthesiologists classification (ASA, categorised 1–5) [25], T (1–4) and N (0–2) stage and tumour location (colon/rectum) to get stage adjusted estimates. Estimates without adjusting for the stage (T-stage and N-stage) and comorbidities (CCI and ASA) were produced separately to assess the role of the mediators (this corresponds to the adjustment set without potential mediators).

Five models using all available data were fitted for each outcome and adjustment set (with and without the adjustment for mediators stage and comorbidities): (1) without exposures, (2) with income, (3) with education, (4) with a time-varying effect (i.e., non-proportional hazards) for income (5) with a time-varying effect for education. Models within each adjustment set were compared using the likelihood ratio test to test the statistical significance of the exposure of interest (by comparing model 2–5 to model 1) and test for time-varying effects (by comparing model 4 to model 2 and model 5 to model 3). Based on the likelihood ratio test, the best models for income and education (i.e., with or without time-varying effects) were used to obtain marginal estimates, i.e., standardised survival curves under the least and most deprived groups using the adjustment sets described in the previous paragraph.

Survival estimates and Hazard ratios (HR) of recurrence, cancer-specific mortality and all-cause mortality, were obtained from the models as they provide distinct insights. HRs compare the *rates* of experiencing an outcome across different income or education groups, while survival probabilities indicate the *risk* of experiencing the event within a specific timeframe [28]. Different income and education groups have different covariate distributions that can explain part of the survival differences. The HRs are adjusted for the covariate distribution (the adjustment sets described previously), and it is therefore assumed that the relative difference in rates is the same within each covariate pattern. However, this is not the case for the survival function since it is an absolute measure and will be different for each covariate pattern. To make fairer comparisons, standardised survival functions were therefore obtained. Different choices can be made in terms of which population is used as the “standard“ population [28]. In the present study, the covariate pattern observed in the most deprived (i.e., lowest income or education group) was used. For the analysis, flexible parametric survival models were fitted by including all patients and adjusting for all covariates. Then, standardised survival estimates were obtained based on these model parameters and using the covariate pattern of the most deprived as the standard. We obtained the standardised survival functions under the highest and under the lowest deprivation, and these can be interpreted as the observed survival of the most deprived and the survival that the most deprived would have if they had belonged to the least deprived group, respectively. HR and standardised survival proportions were calculated at 1, 3 and 5 years for TTR and at 1, 3, 5 and 10 years for CSS and OS. *p* values < 0.05 were considered statistically significant. Statistics were calculated using R (version 4.3.2; R Core Team 2023) [29] and the rstpm2 package [30, 31] was used to produce FPM and calculate HR, rates and survival curves.

## Results

There were 89,107 patients diagnosed with first-time CRC between 2008 and 2021 included in the database. After the exclusion of 18,925 patients with synchronous metastatic disease, 1864 patients without pathological stage information, 7818 where no resection was performed and 505 individuals who were classified as stage 0 in the registry the final cohort contained 59,995 patients where the most advanced tumour (highest T and N stage) was used in cases of multiple tumours diagnosed on the same day (Supplementary Fig. 2). There were 56,204 patients with data on income and 55,584 with data on education. Patients who did not have surgery were older, more comorbid and had lower income and less education than patients who had surgery (Supplementary Table 2).

### Patient and tumour characteristics

The patients in the most deprived income (Q1) or least education (<9 years) groups were older, more comorbid (proportion of ASA3 and ASA4 38.7% vs. 25.2% in most vs. least deprived for income), and more often lived alone. In this group, colon cancer was more common (72.2% vs. 68.1% in least vs. most deprived for income) as were, higher malignancy grade, mucinous features, emergency surgery, more postoperative complications, and less neoadjuvant and adjuvant treatment. In addition, patients with the most deprived income had higher T-stage (18.1% T4 vs. 16.6% in the least deprived) and were more often node-positive. In those with the least education, T3 and node-negative tumours (N0) were more common, whereas vascular and perineural invasion were less common. See Tables 1 (Demographics) and 2 (Tumour characteristics) for details.

**Table 1:** Demographics of resected colorectal cancer patients diagnosed with stage I–III disease in Sweden during the years 2008–2021, by income (in quartiles, Q) and education group (based on the number of years of schooling).

	Income					Education		
	Total	Q1	Q2	Q3	Q4	<9 years	9–12 years	>12 years
Sex								
Male	29,358 (52.2%)	6983 (51.8%)	7287 (52.4%)	7518 (52.5%)	7570 (52.3%)	7788 (53.3%)	9746 (46.7%)	11,512 (57.3%)
Female	26,846 (47.8%)	6499 (48.2%)	6627 (47.6%)	6809 (47.5%)	6911 (47.7%)	6821 (46.7%)	11,133 (53.3%)	8584 (42.7%)
Age								
Median (IQR)	73.0 (65.0–80.0)	75.0 (65.0–82.0)	75.0 (65.0–81.0)	73.0 (65.0–79.0)	70.0 (65.0–76.0)	78.0 (72.0–83.0)	71.0 (63.0–78.0)	70.0 (62.0–77.0)
ASA								
1	7606 (13.5%)	1413 (10.5%)	1736 (12.5%)	2057 (14.4%)	2400 (16.6%)	1041 (7.1%)	2810 (13.5%)	3717 (18.5%)
2	29,201 (52.0%)	6554 (48.6%)	6885 (49.5%)	7559 (52.8%)	8203 (56.6%)	7171 (49.1%)	11,001 (52.7%)	10,748 (53.5%)
3	16,857 (30.0%)	4748 (35.2%)	4594 (33.0%)	4113 (28.7%)	3402 (23.5%)	5515 (37.8%)	6197 (29.7%)	4893 (24.3%)
4	1527 (2.7%)	478 (3.5%)	463 (3.3%)	340 (2.4%)	246 (1.7%)	584 (4.0%)	519 (2.5%)	389 (1.9%)
5	31 (0.1%)	11 (0.1%)	9 (0.1%)	6 (0.0%)	5 (0.0%)	16 (0.1%)	12 (0.1%)	3 (0.0%)
Missing	982 (1.7%)	278 (2.1%)	227 (1.6%)	252 (1.8%)	225 (1.6%)	282 (1.9%)	340 (1.6%)	346 (1.7%)
CCI								
Median (IQR)	0.0 (0.0–2.0)	0.0 (0.0–2.0)	0.0 (0.0–2.0)	0.0 (0.0–2.0)	0.0 (0.0–1.0)	0.0 (0.0–2.0)	0.0 (0.0–2.0)	0.0 (0.0–2.0)
Civil								
Alone	22,134 (39.4%)	8612 (63.9%)	6378 (45.8%)	3936 (27.5%)	3208 (22.2%)	6517 (44.6%)	8583 (41.1%)	6728 (33.5%)
Not alone	34,070 (60.6%)	4870 (36.1%)	7536 (54.2%)	10,391 (72.5%)	11,273 (77.8%)	8092 (55.4%)	12,296 (58.9%)	13,368 (66.5%)
Education								
-9 yr	14,609 (26.0%)	5448 (40.4%)	4486 (32.2%)	3077 (21.5%)	1598 (11.0%)			
9 yr–12 yr	20,877 (37.1%)	5054 (37.5%)	5722 (41.1%)	5626 (39.3%)	4475 (30.9%)			
12 yr-	20,095 (35.8%)	2510 (18.6%)	3644 (26.2%)	5578 (38.9%)	8363 (57.8%)			
Missing	623 (1.1%)	470 (3.5%)	62 (0.4%)	46 (0.3%)	45 (0.3%)			

There were 56,204 patients with information on income and 55,584 with information on education.

*ASA* American Society of Anesthesiologists classification, *CCI* Charlson Comorbidity Index, *IQR* interquartile range, *Q* quartile.

### Unadjusted estimates of mortality

The median follow-up until censoring or death was 4.9 years. Unadjusted estimates for OS in the form of Kaplan–Meier curves along with the number at-risk table to illustrate censoring for OS are presented in Supplementary Figs. 3 and 4. In these unadjusted analyses, the most deprived had worse survival than the least deprived.

**Table 2:** Tumour and treatment characteristics by income (in quartiles, Q) and education groups (based on the number of years of schooling) for resected colorectal cancer patients diagnosed with stage I–III disease in Sweden during the years 2008–2021.

	Income					Education		
	Total	Q1	Q2	Q3	Q4	<9 years	9–12 years	>12 years
Location								
Right	23,394 (41.6%)	5817 (43.1%)	6049 (43.5%)	5904 (41.2%)	5624 (38.8%)	6740 (46.1%)	8603 (41.2%)	7783 (38.7%)
Left	16,044 (28.5%)	3924 (29.1%)	3837 (27.6%)	4043 (28.2%)	4240 (29.3%)	3935 (26.9%)	5863 (28.1%)	6059 (30.2%)
Rectum	16,708 (29.7%)	3731 (27.7%)	4006 (28.8%)	4365 (30.5%)	4606 (31.8%)	3919 (26.8%)	6388 (30.6%)	6236 (31.0%)
Missing	58 (0.1%)	10 (0.1%)	22 (0.2%)	15 (0.1%)	11 (0.1%)	15 (0.1%)	25 (0.1%)	18 (0.1%)
T stage								
T0–2	14,792 (26.3%)	3203 (23.8%)	3442 (24.7%)	3883 (27.1%)	4264 (29.4%)	3512 (24.0%)	5468 (26.2%)	5678 (28.3%)
T3	31,610 (56.2%)	7837 (58.1%)	7959 (57.2%)	8018 (56.0%)	7796 (53.8%)	8561 (58.6%)	11,641 (55.8%)	11,040 (54.9%)
T4	9742 (17.3%)	2434 (18.1%)	2496 (17.9%)	2407 (16.8%)	2405 (16.6%)	2528 (17.3%)	3750 (18.0%)	3346 (16.7%)
Missing	60 (0.1%)	8 (0.1%)	17 (0.1%)	19 (0.1%)	16 (0.1%)	8 (0.1%)	20 (0.1%)	32 (0.2%)
N stage								
N0	34,169 (60.8%)	8176 (60.6%)	8570 (61.6%)	8687 (60.6%)	8736 (60.3%)	9115 (62.4%)	12,753 (61.1%)	11,925 (59.3%)
N1	14,523 (25.8%)	3422 (25.4%)	3483 (25.0%)	3762 (26.3%)	3856 (26.6%)	3637 (24.9%)	5320 (25.5%)	5407 (26.9%)
N2	7512 (13.4%)	1884 (14.0%)	1861 (13.4%)	1878 (13.1%)	1889 (13.0%)	1857 (12.7%)	2806 (13.4%)	2764 (13.8%)
Stage^a^								
I	12,144 (21.6%)	2665 (19.8%)	2861 (20.6%)	3172 (22.1%)	3446 (23.8%)	2944 (20.2%)	4504 (21.6%)	4585 (22.8%)
II	22,025 (39.2%)	5511 (40.9%)	5709 (41.0%)	5515 (38.5%)	5290 (36.5%)	6171 (42.2%)	8249 (39.5%)	7340 (36.5%)
III	22,035 (39.2%)	5306 (39.4%)	5344 (38.4%)	5640 (39.4%)	5745 (39.7%)	5494 (37.6%)	8126 (38.9%)	8171 (40.7%)
Malignancy grade								
Low	43,002 (76.5%)	10,177 (75.5%)	10,541 (75.8%)	11,000 (76.8%)	11,284 (77.9%)	10,970 (75.1%)	15,937 (76.3%)	15,618 (77.7%)
High	10,678 (19.0%)	2671 (19.8%)	2795 (20.1%)	2652 (18.5%)	2560 (17.7%)	3005 (20.6%)	4035 (19.3%)	3528 (17.6%)
Missing	2524 (4.5%)	634 (4.7%)	578 (4.2%)	675 (4.7%)	637 (4.4%)	634 (4.3%)	907 (4.3%)	950 (4.7%)
Vascular invasion								
No	38,026 (67.7%)	8982 (66.6%)	9408 (67.6%)	9795 (68.4%)	9841 (68.0%)	9875 (67.6%)	14,201 (68.0%)	13,539 (67.4%)
Yes	15,249 (27.1%)	3506 (26.0%)	3773 (27.1%)	3905 (27.3%)	4065 (28.1%)	3760 (25.7%)	5661 (27.1%)	5647 (28.1%)
Missing	2929 (5.2%)	994 (7.4%)	733 (5.3%)	627 (4.4%)	575 (4.0%)	974 (6.7%)	1017 (4.9%)	910 (4.5%)
Perineural invasion								
No	42,201 (75.1%)	9673 (71.7%)	10,458 (75.2%)	10,956 (76.5%)	11,114 (76.7%)	10,769 (73.7%)	15,789 (75.6%)	15,188 (75.6%)
Yes	8912 (15.9%)	2070 (15.4%)	2199 (15.8%)	2249 (15.7%)	2394 (16.5%)	2165 (14.8%)	3316 (15.9%)	3334 (16.6%)
Missing	5091 (9.1%)	1739 (12.9%)	1257 (9.0%)	1122 (7.8%)	973 (6.7%)	1675 (11.5%)	1774 (8.5%)	1574 (7.8%)
Mucinous								
No	44,689 (79.5%)	10,413 (77.2%)	10,967 (78.8%)	11,480 (80.1%)	11,829 (81.7%)	11,232 (76.9%)	16,677 (79.9%)	16,293 (81.1%)
Yes	8508 (15.1%)	2139 (15.9%)	2207 (15.9%)	2177 (15.2%)	1985 (13.7%)	2411 (16.5%)	3181 (15.2%)	2814 (14.0%)
Missing	3007 (5.4%)	930 (6.9%)	740 (5.3%)	670 (4.7%)	667 (4.6%)	966 (6.6%)	1021 (4.9%)	989 (4.9%)
Neoadjuvant treatment								
No	48,718 (86.7%)	11,836 (87.8%)	12,097 (86.9%)	12,333 (86.1%)	12,452 (86.0%)	12,892 (88.2%)	17,958 (86.0%)	17,317 (86.2%)
Radiotherapy	6118 (10.9%)	1401 (10.4%)	1521 (10.9%)	1615 (11.3%)	1581 (10.9%)	1547 (10.6%)	2337 (11.2%)	2171 (10.8%)
Radio-chemo	755 (1.3%)	114 (0.8%)	159 (1.1%)	230 (1.6%)	252 (1.7%)	81 (0.6%)	342 (1.6%)	328 (1.6%)
Chemo	613 (1.1%)	131 (1.0%)	137 (1.0%)	149 (1.0%)	196 (1.4%)	89 (0.6%)	242 (1.2%)	280 (1.4%)
Emergency surgery								
Elective	49,943 (88.9%)	11,523 (85.5%)	12,264 (88.1%)	12,945 (90.4%)	13,211 (91.2%)	12,698 (86.9%)	18,579 (89.0%)	18,145 (90.3%)
Emergency	6233 (11.1%)	1953 (14.5%)	1642 (11.8%)	1374 (9.6%)	1264 (8.7%)	1900 (13.0%)	2289 (11.0%)	1945 (9.7%)
Missing	28 (0.0%)	6 (0.0%)	8 (0.1%)	8 (0.1%)	6 (0.0%)	11 (0.1%)	11 (0.1%)	6 (0.0%)
Postoperative complications								
No	39,105 (69.6%)	9112 (67.6%)	9632 (69.2%)	10,061 (70.2%)	10,300 (71.1%)	9865 (67.5%)	14,635 (70.1%)	14,212 (70.7%)
Yes	16,813 (29.9%)	4307 (31.9%)	4209 (30.3%)	4189 (29.2%)	4108 (28.4%)	4675 (32.0%)	6138 (29.4%)	5776 (28.7%)
Missing	286 (0.5%)	63 (0.5%)	73 (0.5%)	77 (0.5%)	73 (0.5%)	69 (0.5%)	106 (0.5%)	108 (0.5%)
Adjuvant treatment								
No	43,969 (78.2%)	11,173 (82.9%)	11,150 (80.1%)	10,979 (76.6%)	10,667 (73.7%)	12,542 (85.9%)	16,112 (77.2%)	14,783 (73.6%)
Chemotherapy	12,198 (21.7%)	2301 (17.1%)	2753 (19.8%)	3336 (23.3%)	3808 (26.3%)	2060 (14.1%)	4746 (22.7%)	5305 (26.4%)
Radio-chemo	37 (0.1%)	8 (0.1%)	11 (0.1%)	12 (0.1%)	6 (0.0%)	7 (0.0%)	21 (0.1%)	8 (0.0%)

There were 56,204 patients with information on income and 55,584 with information on education.

^a^TNM stage were classified as stage III if any T and N1-2M0, stage II if missing T and N0M0 or T3–4 and unknown N or N0 and unknown M or M0, and stage I if T1–2 and unknown N and M or N0 and unknown M.

### Recurrences

Neither income nor education was statistically significantly associated with TTR in the multivariable models. Models with time-varying effects did not yield a better fit and were therefore not used.

There was no difference in the standardised probability of patients being recurrence-free at 5 years (82.2% in income Q1 vs. 82.3% in income Q4), Supplementary Fig. 5 and Table 3. Results were similar when omitting tumour characteristics, Supplementary Fig. 6 and Supplementary Table 3, and comorbidities, another potential mediator, from the model, Supplementary Fig. 7 and Supplementary Table 4.

**Table 3:** Hazard ratios of CSS and OS for least and most deprived by income (in quartiles) and education (based on the number of years of schooling) at 1, 3, 5 and 10 years for resected colorectal cancer patients diagnosed with stage I–III disease in Sweden during the years 2008–2021.

	HR (95%CI)	HR (95%CI)	HR (95%CI)	HR (95%CI)
	1-year CSS	3-year CSS	5-year CSS	10-year CSS
Income				
Q4 vs. Q1	0.76 (0.71–0.82)	0.87 (0.83–0.91)	0.93 (0.87–0.99)	1.01 (0.91–1.12)
Education				
>12 yr vs. <9 yr	0.86 (0.80–0.91)	0.92 (0.88–0.96)	0.95 (0.90–1.00)	0.99 (0.90–1.08)
	**1-year OS**	**3-year OS**	**5-year OS**	**10-year OS**
Income				
Q4 vs. Q1	0.78 (0.73–0.82)	0.81 (0.78–0.84)	0.82 (0.79–0.86)	0.85 (0.80–0.90)
Education				
>12 yr vs. <9 yr	0.90 (0.86–0.95)	0.87 (0.84–0.9)	0.84 (0.81–0.87)	0.81 (0.76–0.85)

Standardised to the distribution of civil status, year of diagnosis, sex, age, CCI, ASA, T and N stage and tumour location of the most deprived.

*95%CI* 95% confidence interval, *CSS* cancer-specific survival, *HR* hazard ratio, *OS* overall survival, *Q* quartile.

Similarly, the standardised probability of being recurrence-free at 5 years in the least and the most educated groups was 82.5% and 82.4%, respectively, Supplementary Fig. 5 and Table 3. Results were similar when omitting tumour characteristics, Supplementary Fig. 6 and Supplementary Table 3, and comorbidities from the model, Supplementary Fig. 7 and Supplementary Table 4. The HR for recurrence rates was 0.99 (95%CI 0.92–1.05) for income Q4 vs. Q1 and 1.01 (95%CI 0.95–1.07) for >12 years vs. <9 years of education. Similar results were seen when omitting tumour characteristics and comorbidities, Supplementary Tables 5 and 6.

### Cancer-specific survival

Both income and education were statistically significantly associated with cancer-specific mortality within their respective models (Likelihood ratio test *p* < 0.001 for both income and education). Time-varying effects for income and education were also statistically significant (Likelihood ratio test *p* < 0.001 for both income and education) and so the models with time-varying effects were used to calculate adjusted survival and HR for CSS.

**Fig. 1:**
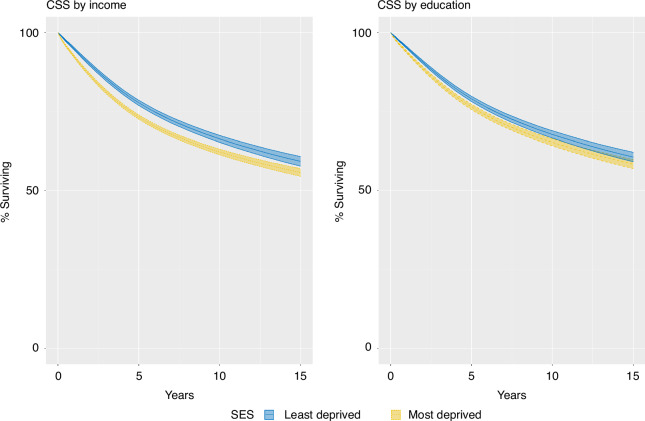
Standardised cancer-specific survival for resected colorectal cancer patients with stage I–III disease by deprivation group. Left panel: Income, Right panel: Education. Adjusted to the distribution of civil status, year of diagnosis, sex, age, CCI, ASA, T and N stage and tumour location of the most deprived. Least deprived: Income Q4. Most deprived: Income Q1. Most educated: >12 years of education. Least educated: <9 years of education.

The 5-year standardised CSS was 73.2% (95%CI 72.6–73.9) in income Q1, and the standardised CSS estimate had they been in the least deprived income group, Q4, was 77.8% (95%CI 76.9–78.6), resulting in a difference of 4.6 percentage points between income groups, Fig. 1 and Table 3. Omitting tumour characteristics from the model, the difference in 5-year standardised CSS was 5.3% between the most and least deprived income groups, Supplementary Fig. 8 and Supplementary Table 3. Omitting tumour characteristics and comorbidities from the model, the difference in 5-year standardised CSS was 6.1% between the most and least deprived income groups, Supplementary Fig. 9 and Supplementary Table 4.

Similarly, the 5-year standardised CSS by education were 76.5% (95%CI 75.6-77.3) in those with <9 years of education and 79% (95%CI 78.2–79.9) with >12 years of education, resulting in a 2.5 percentage points difference between education groups, Fig. 1 and Table 3. Omitting tumour characteristics from the model, the difference in 5-year standardised CSS was 2.9% between the least and most educated, Supplementary Fig. 8 and Supplementary Table 3. Omitting tumour characteristics and comorbidities from the model, the difference in 5-year standardised CSS was 3.3% between the least and most educated, Supplementary Fig. 9 and Supplementary Table 4. Overall, differences in CSS between the groups increased during the first 5 years and then decreased (for income group) or stabilised (for education groups).

The HR for CSS at 1 year was 0.76 (95%CI 0.71–0.82) in income Q4 vs. Q1 and 0.86 (95%CI 0.80–0.91) for >12 years vs. <9 years of education. HRs at 3, 5 and 10 years moved towards 1 over time, Table 4. When omitting tumour characteristics, the 1-year HR was 0.74 (95%CI 0.68–0.79) in income Q4 vs. Q1 and 0.84 (95%CI 0.78–0.89) for >12 years vs. <9 years of education, Supplementary Table 5. Differences between income and education groups were increased when omitting stage and comorbidities from the models, with the 1-year HR at 0.70 (95%CI 0.65–0.75) for income Q4 vs. Q1 and 0.81 (95%CI 0.76–0.86) for >12 years vs. <9 years of education, Supplementary Table 6.

**Table 4:** Standardised survival estimates for TTR, CSS and OS for the least and most deprived by income (in quartiles) and education (based on the number of years of schooling) at 1, 3, 5 and 10 years for resected colorectal cancer patients diagnosed with stage I–III disease in Sweden during the years 2008–2021.

	% (95%CI)	% (95%CI)	% (95%CI)	% (95%CI)
	1-year TTR	3-year TTR	5-year TTR	
Income				
Q1	94.2% (93.9–94.5)	84.9% (84.4–85.5)	82.2% (81.5–82.8)	
Q4	94.2% (93.9–94.6)	85.1% (84.4–85.8)	82.3% (81.5–83.2)	
Education				
<9 yr	94.3% (93.9–94.6)	85.2% (84.5–85.9)	82.5% (81.7–83.3)	
>12 yr	94.2% (94.0–94.8)	85.1% (84.5–85.9)	82.4% (81.5–83.1)	
	**1-year CSS**	**3-year CSS**	**5-year CSS**	**10-year CSS**
Income				
Q1	92.4% (92.0–92.8)	81.2% (80.6–81.8)	73.2% (72.6–73.9)	62.2% (61.3–63.1)
Q4	94.9% (94.5–95.2)	85.3% (84.6–86)	77.8% (76.9–78.6)	66.4% (65.2–67.5)
Education				
<9 yr	93.8% (93.4–94.1)	84.0% (83.3–84.6)	76.5% (75.6–77.3)	65.3% (64.2–66.4)
>12 yr	95.2% (94.9–95.6)	86.3% (85.6–86.9)	79.0% (78.2–79.9)	67.8% (66.6–68.9)
	**1-year OS**	**3-year OS**	**5-year OS**	**10-year OS**
Income				
Q1	90.1% (89.6–90.5)	75.1% (74.5–75.7)	63.5% (62.9–64.1)	42.9% (42.1–43.6)
Q4	92.8% (92.3–93.2)	80.2% (79.5–81.0)	70.0% (69.1–70.8)	50.4% (49.4–51.4)
Education				
<9 yr	92.3% (91.9–92.7)	79.2% (78.5–79.9)	67.7% (66.8–68.6)	44.9% (43.8–46.0)
>12 yr	93.4% (93.1–93.8)	81.5% (80.8–82.3)	71.4% (70.5–72.3)	51.0% (49.8–52.2)

Standardised to the distribution of civil status, year of diagnosis, sex, age, CCI, ASA, T and N stage and tumour location of the most deprived.

*95%CI* 95% confidence interval, *CSS* cancer-specific survival, *OS* overall survival, *Q* quartile, *TTR* time to recurrence, recurrence-free proportion reported.

### Overall survival

Both income and education were statistically significantly associated with all-cause mortality within their respective models (Likelihood ratio test *p* < 0.001 for both income and education). Time-varying effects for income and education were also statistically significant (Likelihood ratio test *p* 0.001 and <0.001 for income and education, respectively) and so the models with time-varying effects were used to calculate adjusted survival and HR for OS.

**Fig. 2:**
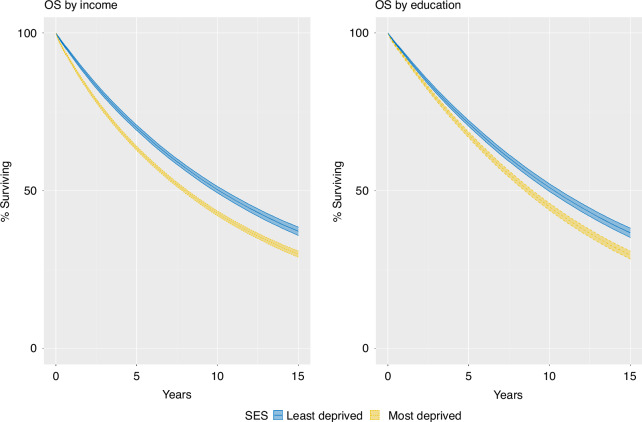
Standardised overall survival for resected colorectal cancer patients with stage I–III disease by deprivation group. Left panel: Income, Right panel: Education. Adjusted to the distribution of civil status, year of diagnosis, sex, age, CCI, ASA, T and N stage and tumour location of the most deprived. Least deprived: Income Q4. Most deprived: Income Q1. Most educated: >12 years of education. Least educated: <9 years of education.

The standardised 5-year OS by income were 63.5% (95%CI 62.9–64.1) in income Q1 and 70.0% (95%CI 69.1–70.8) in income Q4, Fig. 2 and Table 3. Omitting tumour characteristics from the model, the difference in 5-year standardised OS was 6.9% between the least and most deprived income groups, Supplementary Fig. 10 and Supplementary Table 3. Omitting tumour characteristics and comorbidities from the model, the difference in 5-year standardised OS was 8.3% between the least and most deprived income group, Supplementary Fig. 11 and Supplementary Table 4.

The standardised 5-year OS by education was 67.7% (95%CI 66.8–68.6) in those with <9 years of education and 71.4% (95%CI 70.5–72.3) for >12 years of education, Fig. 2 and Table 3. The differences in OS between groups increased with time, and contrary to what was seen with CSS there was no plateau. Omitting tumour characteristics from the model, the difference in 5-year standardised OS was 3.8% between the least and most educated, Supplementary Fig. 10 and Supplementary Table 3. Omitting tumour characteristics and comorbidities from the model, the difference in 5-year standardised OS was 4.5% between the least and most educated, Supplementary Fig. 11 and Supplementary Table 4.

The adjusted HR for OS at 1 year were 0.82 (95%CI 0.79–0.86) in income Q4 vs. Q1 and 0.84 (95%CI 0.81–0.87) for >12 years vs. <9 years of education. HRs at 3, 5 and 10 years remained stable for income and decreased for education, Table 3. The 1-year HRs were 0.77 (95%CI 0.72–0.81) for income Q4 vs. Q1 and 0.90 (95%CI 0.85–0.95) for >12 years vs. <9 years of education when omitting tumour characteristics, Supplementary Table 5. When omitting tumour and comorbidities, differences between income groups were larger with a 1-year HR at 0.72 (95%CI 0.67–0.76) for income Q4 vs. Q1 and 0.86 (95%CI 0.81–0.90) for >12 years vs. <9 years of education, Supplementary Table 6.

## Discussion

In this nationwide cohort study of 59,995 CRC patients with curatively treated stage I–III disease, we show that differences in survival by SEP persist in a modern material. The most deprived (based on income) have 6.5% lower standardised 5-year overall survival. The biggest differences between income and education groups were seen for standardised OS, while there were smaller differences for standardised CSS between income groups. However, differences between the least and most deprived remained above 4 percentage points during follow-up. Interestingly, there was no difference in time to recurrence after adjusting for demographic and tumour factors. This suggests that the survival differences between socioeconomic groups are not necessarily driven by an increased risk of recurrence in the most deprived, besides differences in stage and comorbidities.

The standardised CSS comparing the least to the most deprived in terms of income decreased after 5 years, suggesting that complications from treatment and early recurrences could be partly responsible for the larger early difference between deprivation groups. The difference in standardised OS between those with most and least education increased with time, suggesting that education matters more with time since diagnosis. Income is thought to measure the ability to maintain a healthier lifestyle and affect psychosocial determinants of health, which seem more important in short-term survival after cancer diagnosis [32, 33]. Education is associated with health awareness and the ability to access health care and appears to be more important in long-term survival [33, 34].

CSS captures mortality related to CRC diagnosis, and TTR captures recurrences in the absence of death which might explain the lack of an effect on TTR despite previously explored treatment differences [12]. The cancer care may be equal but unable to compensate for preexisting differences between the groups. Overall, the most deprived patients may be at a higher risk of non-related cancer death due to poorer health, which might compete with the risk of recurrences. There might also be a difference in survival after recurrences explaining part of the differences in CSS between socioeconomic groups. Another explanation could be that more advanced metastatic disease upon diagnosis of recurrence in the most deprived, paralleling the observed more advanced stage at diagnosis. Other potential explanations are lower treatment tolerance or worse general health increasing the risk of complications in the most deprived.

We have previously shown that there are differences in treatment in non-metastatic colorectal cancer according to SEP, even after adjusting for the differences in age, comorbidities, tumour location and staging [35], a difference that is also present in patients with recurrences. Recent studies have shown differences in the treatment and survival of synchronous metastatic disease between men and women [36], access to surgery for metastatic disease between parts of the country, SEP [37–39], and differences in loss of life expectancy by income [13].

Patients in the most deprived income quartile had a 6.5% lower standardised OS at 5 years, which echoes results from England where relative survival was investigated [17]. In a previous Swedish study of rectal cancer patients diagnosed in 1995–2005, the HR of death in resected patients comparing income Q1 vs. Q4 was 1.35 (0.74 if reversing the ratio) suggesting that disparities have not decreased over time [16].

### Strengths and limitations

The SCRCR covers at least 99% of all diagnosed CRC in Sweden and was recently validated [35, 40]. All patients who had surgery for a non-metastasised CRC were included resulting in a large sample size reflective of practice in Sweden, and likely generalisable to other European countries with similar healthcare systems. Data from national registries allow near complete follow-up due to personal identification numbers allowing linkage of information. Previous validation of the national cause of death registry and patient registry indicates that severe disease and malignant diseases are captured to a high degree [41, 42]. Autopsy rates in Sweden are low which could lead to missed recurrences [43]. However, there is no apparent SEP-dependent difference in the correctness of cause of death registration in Sweden [44], but autopsy rates have not been evaluated in relation to SEP. Previous studies on SEP and CRC were performed before the national care guidelines and do not reflect practice today. This study included more recently diagnosed patients, but given the follow-up needed, it is difficult to evaluate discrepancies in real time.

The analysis is limited to what is registered and the reasons and causes for differences cannot be ascertained. The individual part of disposable household income was chosen over individual disposable income to capture income deprivation for those not working or those working and supplying others. Previous research has shown that the most deprived have worse health overall and survival in the general population [45]. Information on tobacco use and performance status was not available, which could have an impact on survival and eligibility for treatment. The missing data in this setting would be challenging to impute, e.g. imputing the education status of individuals with missing data based on the observed data requires strong assumptions. This could lead to biased imputed values and potential misclassification in education groups and, consequently, biased estimates of interest [46]. National screening for CRC started in 2023 after the patients were included in this study but regional screening in one healthcare region has been running since 2008. In the future, screening may impact the rate of emergency surgery, the stage distribution and possibly reduce the difference in recurrence rates. Unfortunately, those with low SEP have lower participation in screening [47].

## Conclusion

In this material spanning 14 years with an average of 5 years of follow-up, there was no difference in TTR, but there were large disparities in both CSS and OS between the least and most deprived income groups and between the most and least educated. Further investigation is required to understand the reasons for these disparities. Efforts to improve screening participation and detection of disease early for the most deprived, and to improve public health for all may impact some disparities.

## Supplementary information

Strobe Reproducability checklist

Supplementary Material

## Data Availability

The data that support the findings of this study are available from the Swedish Colorectal Cancer Registry, Swedish National Board of Welfare, and Statistics Sweden. Restrictions apply to the availability of these data, which were used under license for this study. The statistical code used to produce the results is available from the corresponding author upon reasonable request.
