# Supplementary material for: Impact of socioeconomics on recurrences and survival in non-metastasized colorectal cancer
Source: Br J Cancer. 2025 Oct 4;133(12):1854–63. doi: 10.1038/s41416-025-03224-w (PMC12690107; doi:10.1038/s41416-025-03224-w)
Supplement: Supplementary file 2 — Supplementary Material [file 41416_2025_3224_MOESM2_ESM.pdf]

Impact of socioeconomics on survival in non-metastasized colorectal cancer

Supplementary material

Erik Osterman[1], Elisavet Syriopoulou[2], Anna Martling[1,3], Therese M-L Andersson[2], Caroline Nordenvall[1,3]
1 Department of Molecular Medicine and Surgery, Karolinska Institute, Sweden,
2 Department of Medical Epidemiology and Biostatistics, Karolinska Institute, Sweden
3 Department of Pelvic Cancer, Colorectal Surgery Unit, Karolinska University Hospital, Sweden

Corresponding author: Erik Osterman (ORCID 0000-0003-1621-7872)
Region Uppsala, Akademiska Sjukhuset
Department of Surgery
751 85 Uppsala
Erik.osterman@ki.se

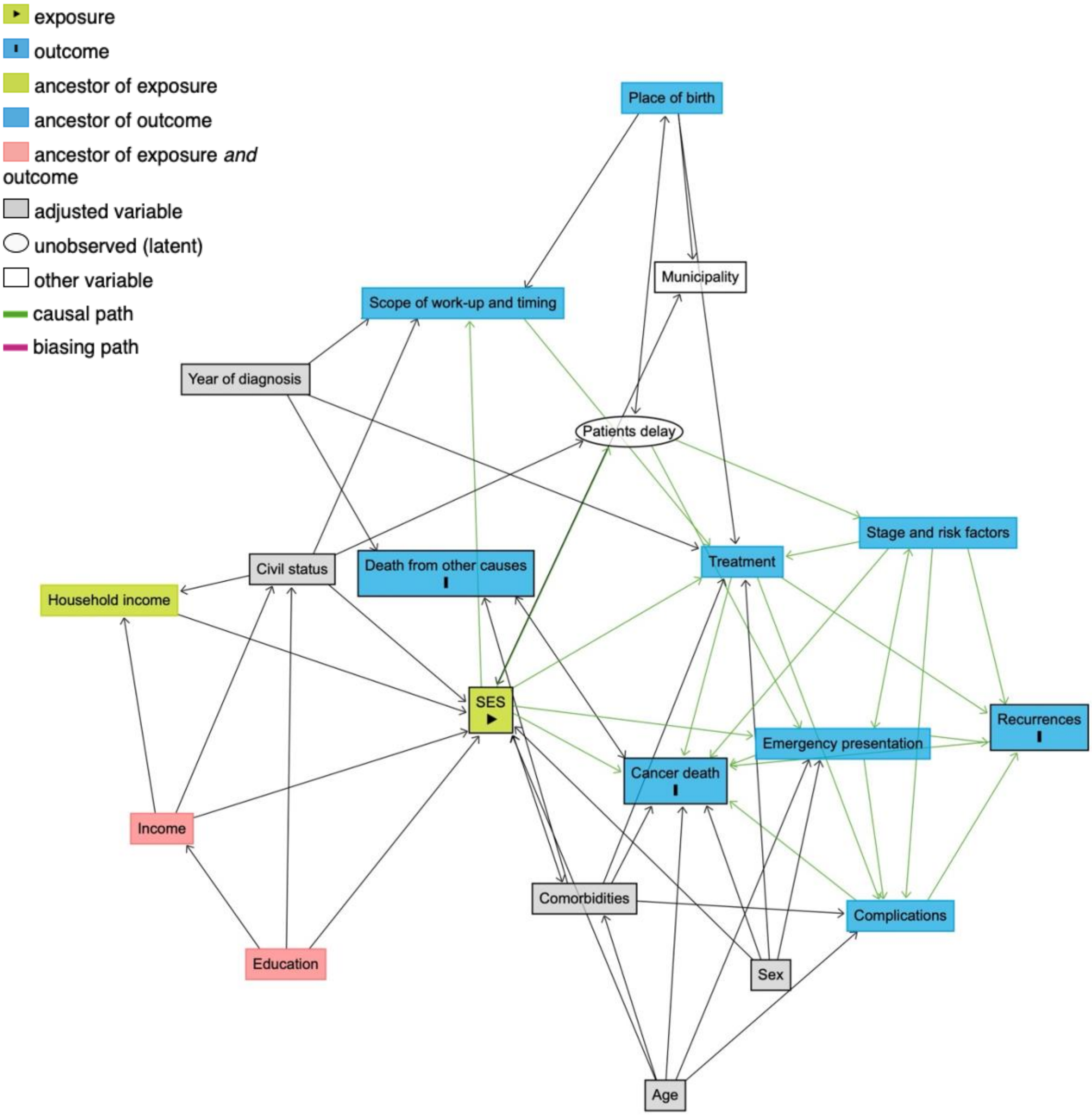

Supplementary Figure 1: Direct Acyclic Graph of variable relationships and adjustments. As stage-adjusted estimates were sought to capture differences besides different stages at diagnosis, additional adjustments for stage and risk factors (pT and pN and tumour location) were made. Place of birth refers to the country of birth which potentially impacts county of residence in Sweden and socioeconomic position.

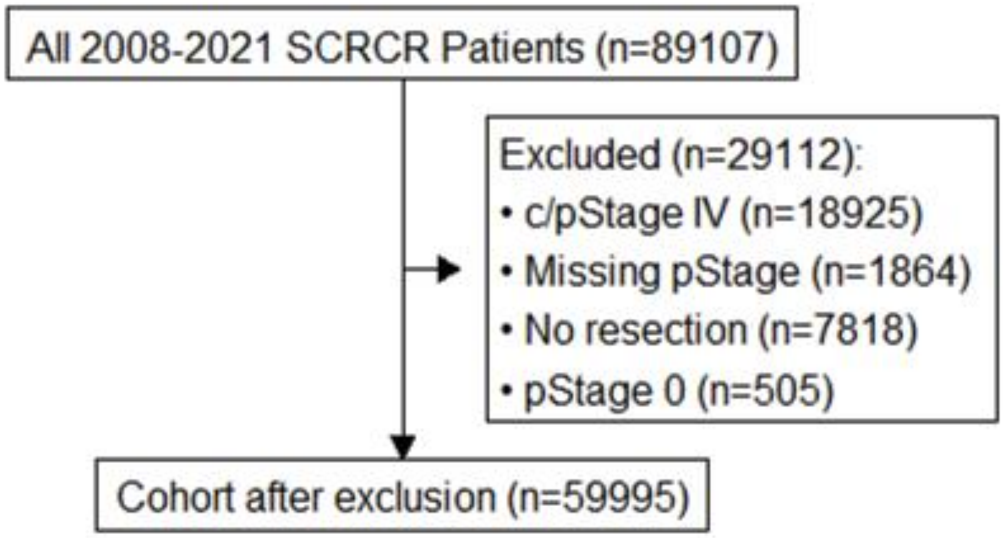

Supplementary Figure 2: Selection of patients to the cohort

Patient data originated from the Colorectal Cancer Database (CRCBaSe), a register-linkage of the Swedish Colorectal Cancer Registry (SCRCR) and national registries at the National Board of Welfare and Statistics Sweden.

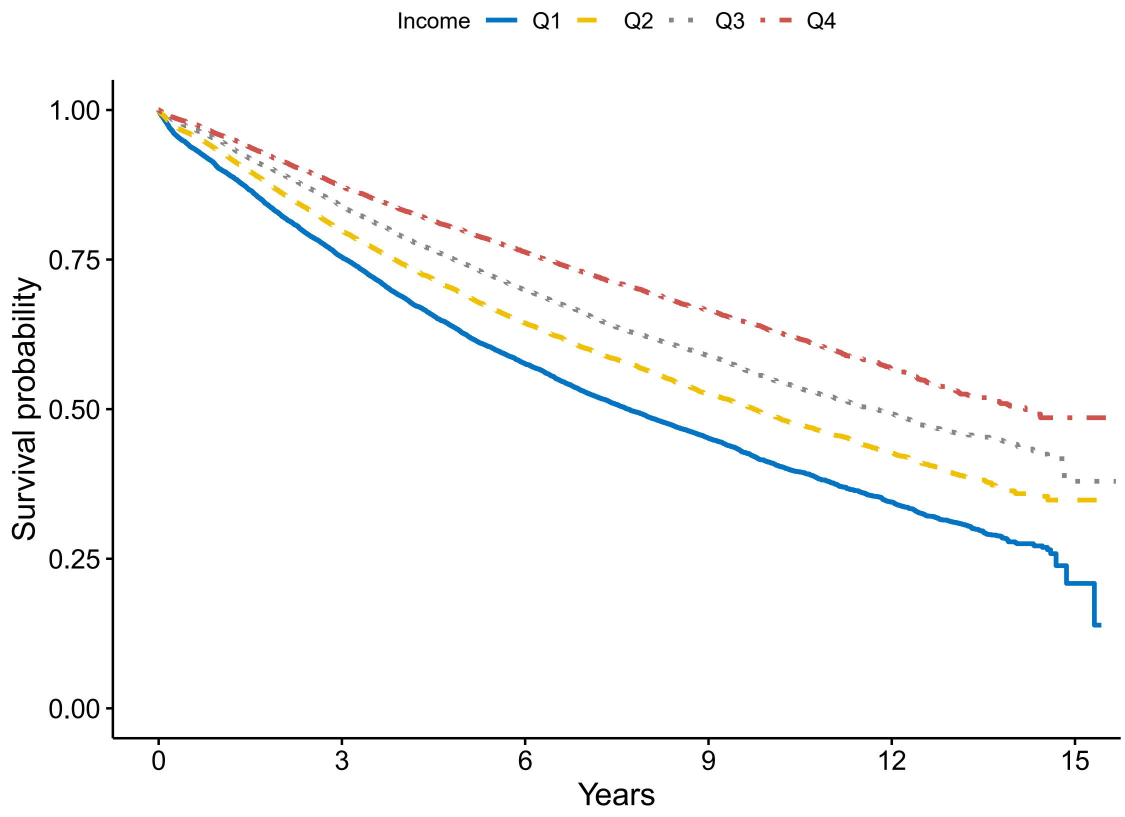

Number at risk

Income	0	3	6	9	12	15
Q1	13482	8992	5469	3029	1166	6
Q2	13914	9307	5548	2950	1116	6
Q3	14327	9702	5667	2989	1093	9
Q4	14481	9808	5726	2774	997	3

Years

Supplementary Figure 3: Kaplan Meier OS by income (in quartiles, Q) for resected colorectal cancer patients diagnosed with stage I-III disease in Sweden during the years 2008-2021. Numbers at risk in table under figure.

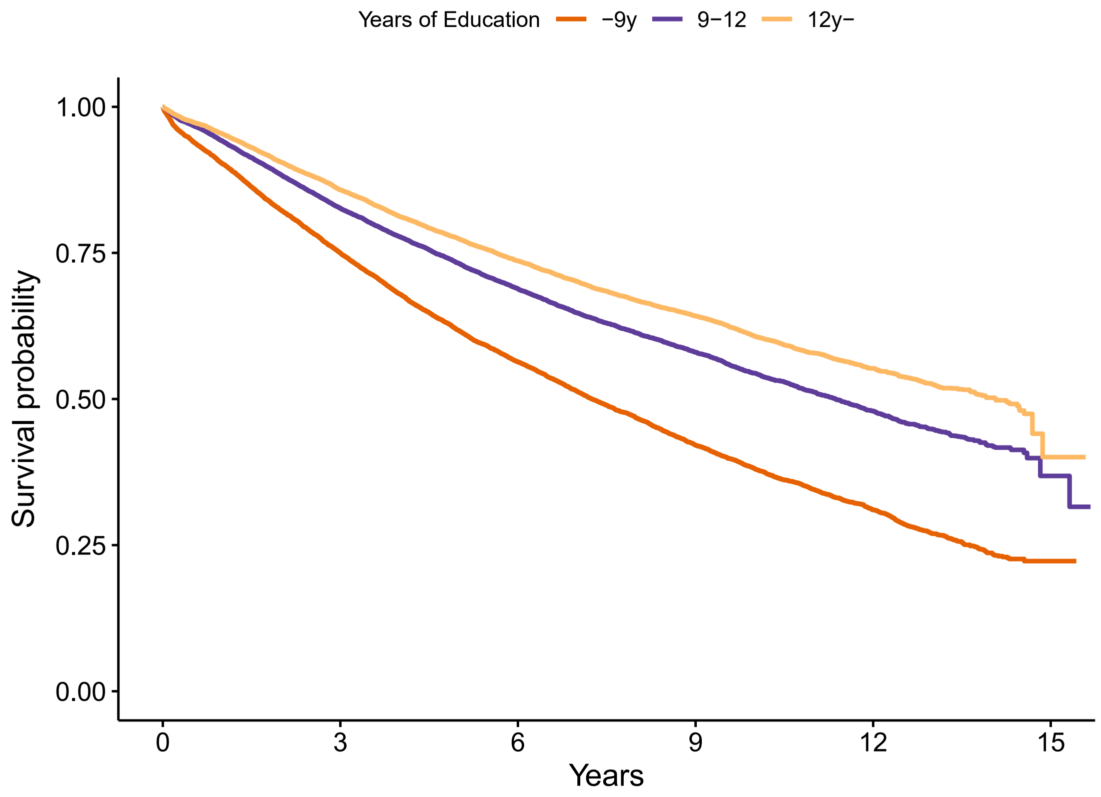

Number at risk

Years of Education	0	3	6	9	12	15
−9y	14609	9582	5640	2910	1073	6
9−12	20879	14118	8343	4399	1604	9
12y−	20096	13734	8218	4329	1660	9

Years

Supplementary Figure 4: Kaplan Meier OS for by education (based on the number of years of schooling) for resected colorectal cancer patients diagnosed with stage I-III disease in Sweden during the years 2008-2021. Numbers at risk in table under figure.

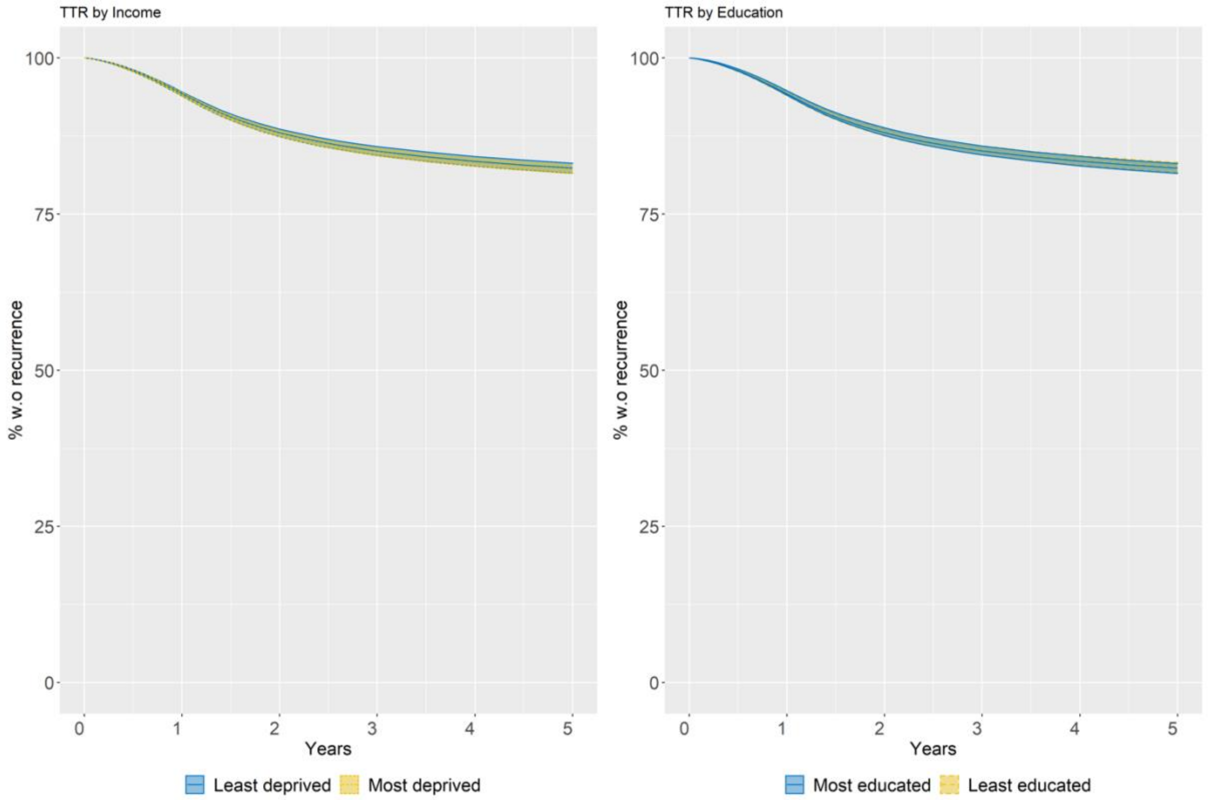

Supplementary Figure 5: Standardised TTR for resected colorectal cancer patients with stage I-III disease by deprivation group.
Left panel: Income, Right panel: Education. Adjusted to the distribution of civil status, year of diagnosis, sex, age, CCI, ASA, T and N stage and tumour location of the most deprived
Least deprived: Income Q4
Most deprived: Income Q1
Most educated: >12 years of education
Least educated: <9 years of education

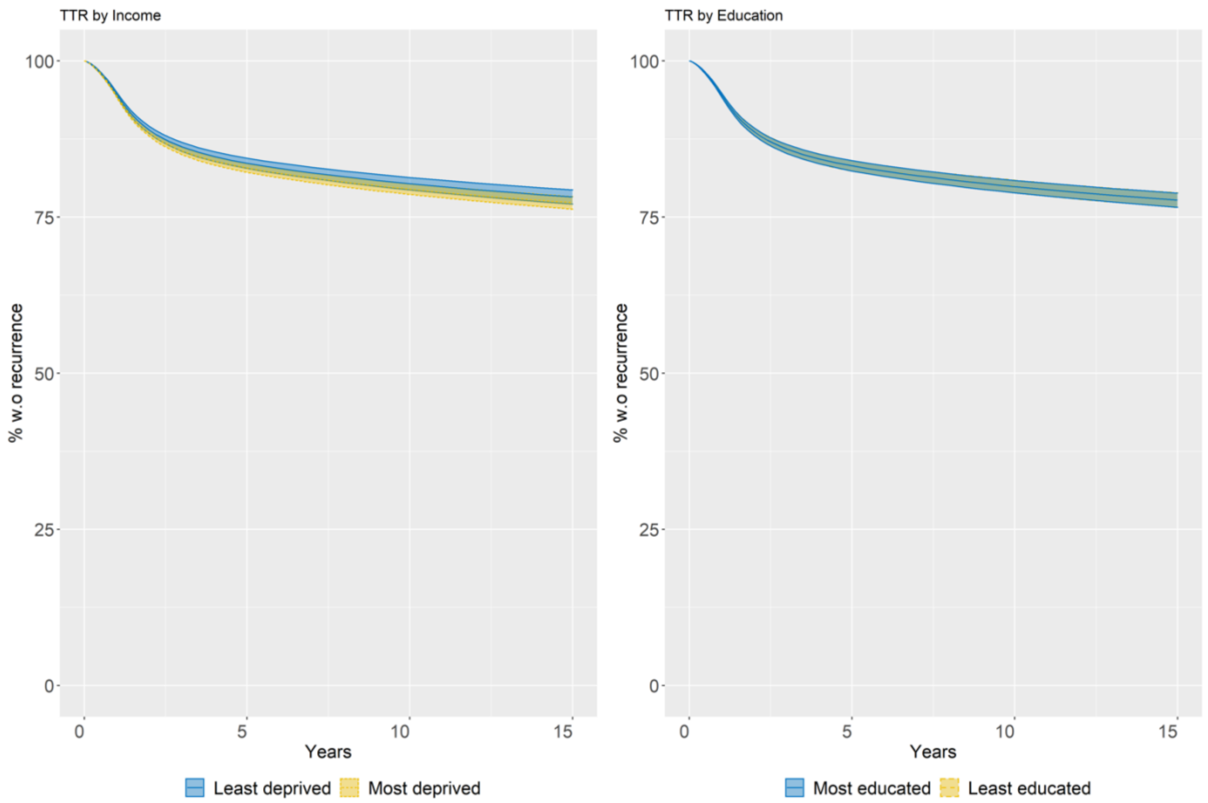

Supplementary Figure 6: Standardised time to recurrence (TTR) for resected colorectal cancer patients with stage I-III disease by deprivation group.
Left panel: Income, Right panel: Education.
Adjusted to the distribution of civil status, year of diagnosis, sex, and age, comorbidities of the most deprived.
Least deprived: Income Q4
Most deprived: Income Q1
Most educated: >12 years of education
Least educated: <9 years of education

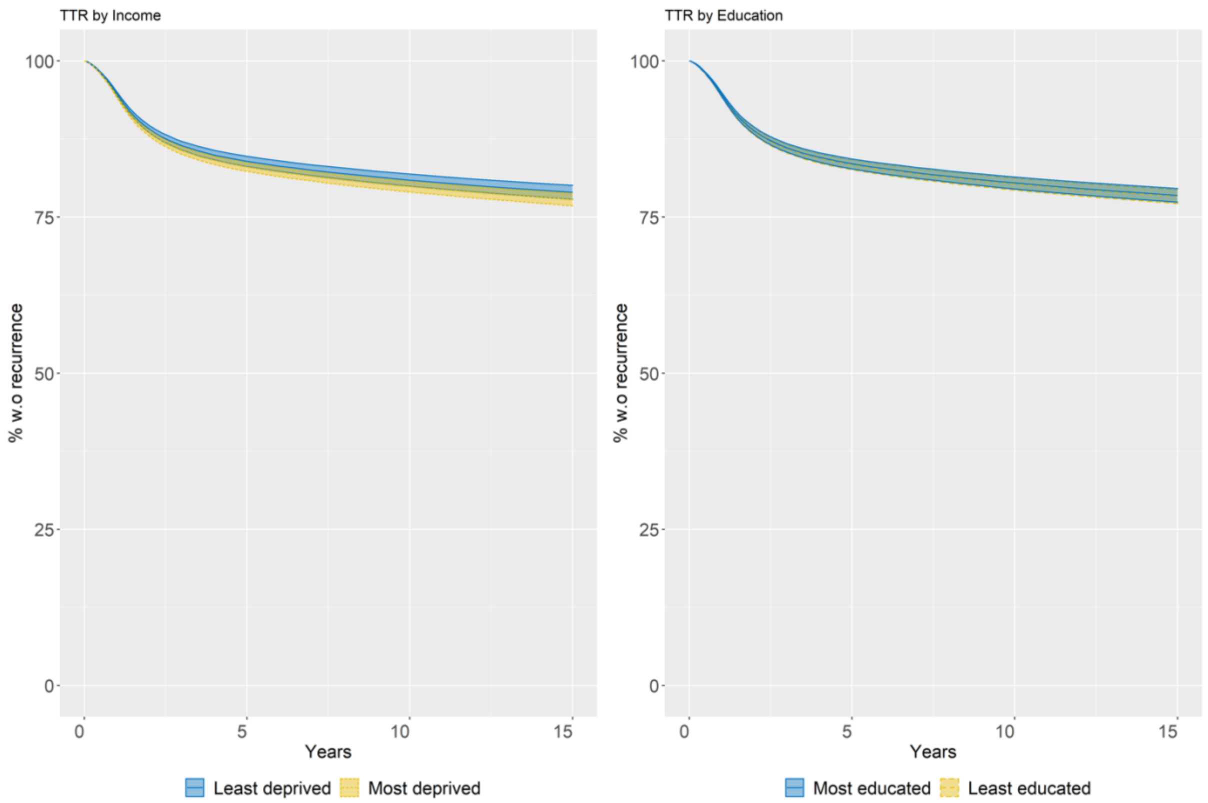

Supplementary Figure 7: Standardised time to recurrence (TTR) for resected colorectal cancer patients with stage I-III disease by deprivation group.

Left panel: Income, Right panel: Education.

Adjusted to the distribution of civil status, year of diagnosis, sex, and age of the most deprived.

Least deprived: Income Q4

Most deprived: Income Q1

Most educated: >12 years of education

Least educated: <9 years of education

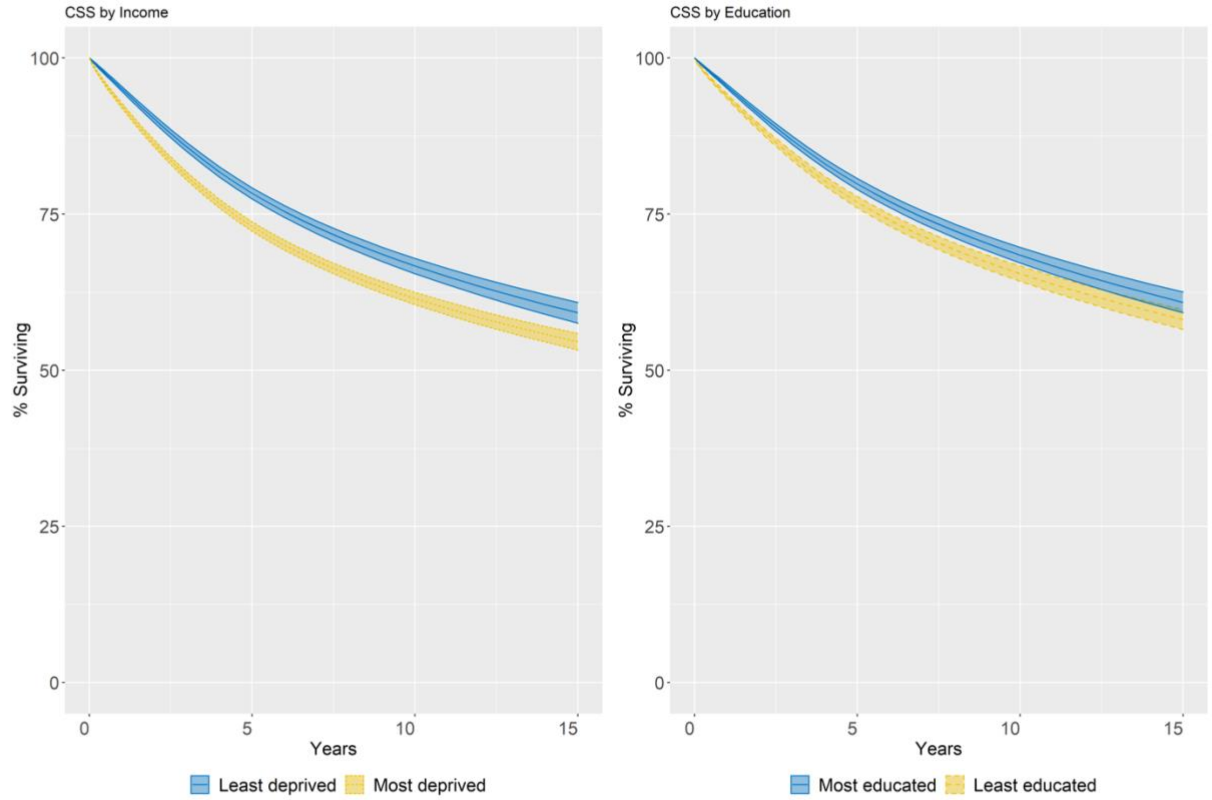

Supplementary Figure 8: Standardised cancer-specific survival (CSS) for resected colorectal cancer patients with stage I-III disease by deprivation group.
Left panel: Income, Right panel: Education.
Adjusted to the distribution of civil status, year of diagnosis, sex, and age, comorbidities of the most deprived.
Least deprived: Income Q4
Most deprived: Income Q1
Most educated: >12 years of education
Least educated: <9 years of education

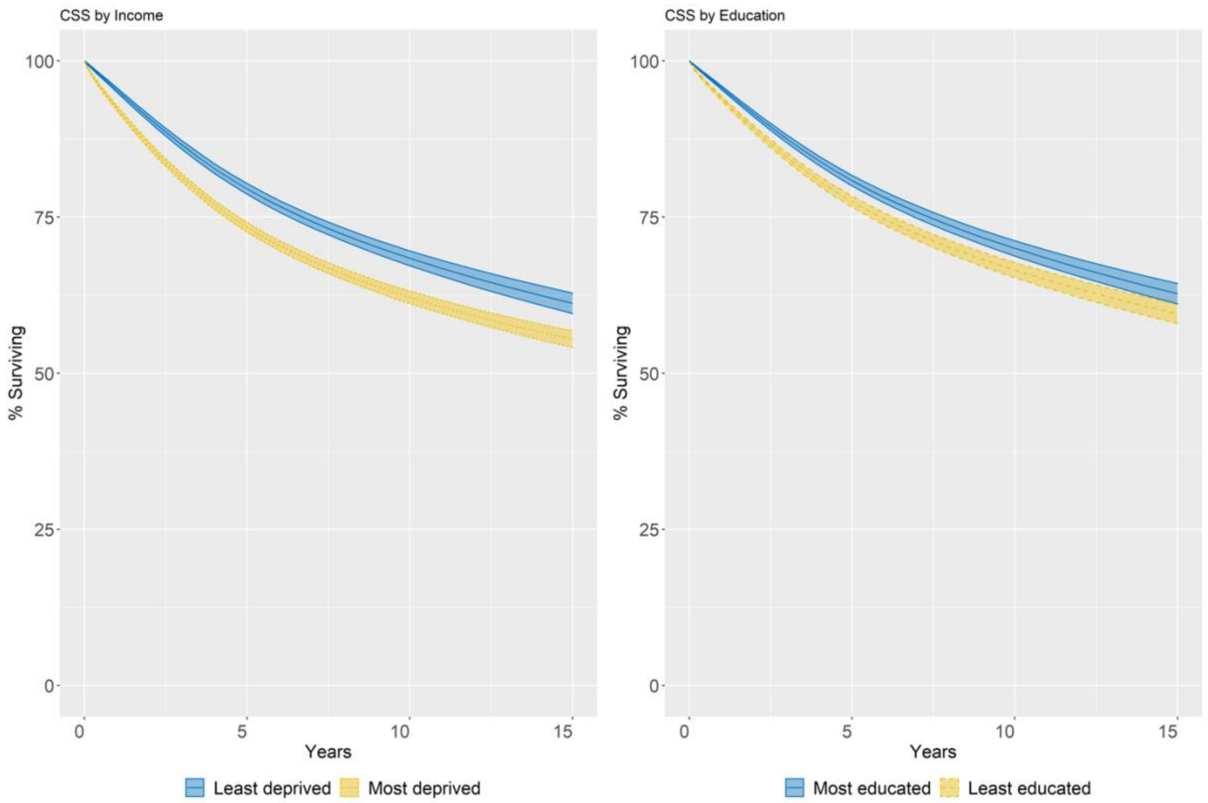

Supplementary Figure 9: Standardised cancer-specific survival (CSS) for resected colorectal cancer patients with stage I-III disease by deprivation group.

Left panel: Income, Right panel: Education.

Adjusted to the distribution of civil status, year of diagnosis, sex, and age of the most deprived.

Least deprived: Income Q4

Most deprived: Income Q1

Most educated: >12 years of education

Least educated: <9 years of education

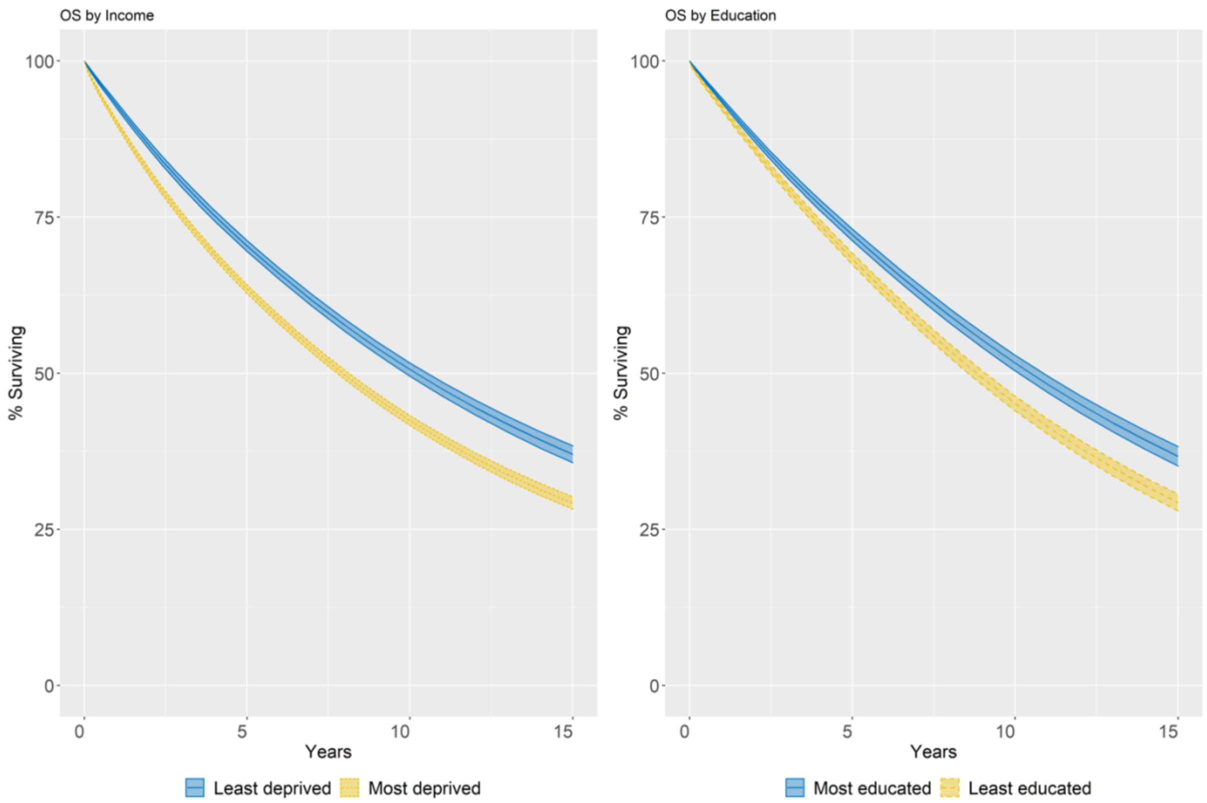

Supplementary Figure 10: Standardised overall survival (OS) for resected colorectal cancer patients with stage I-III disease by deprivation group.
Left panel: Income, Right panel: Education.
Adjusted to the distribution of civil status, year of diagnosis, sex, and age, comorbidities of the most deprived.
Least deprived: Income Q4
Most deprived: Income Q1
Most educated: >12 years of education
Least educated: <9 years of education

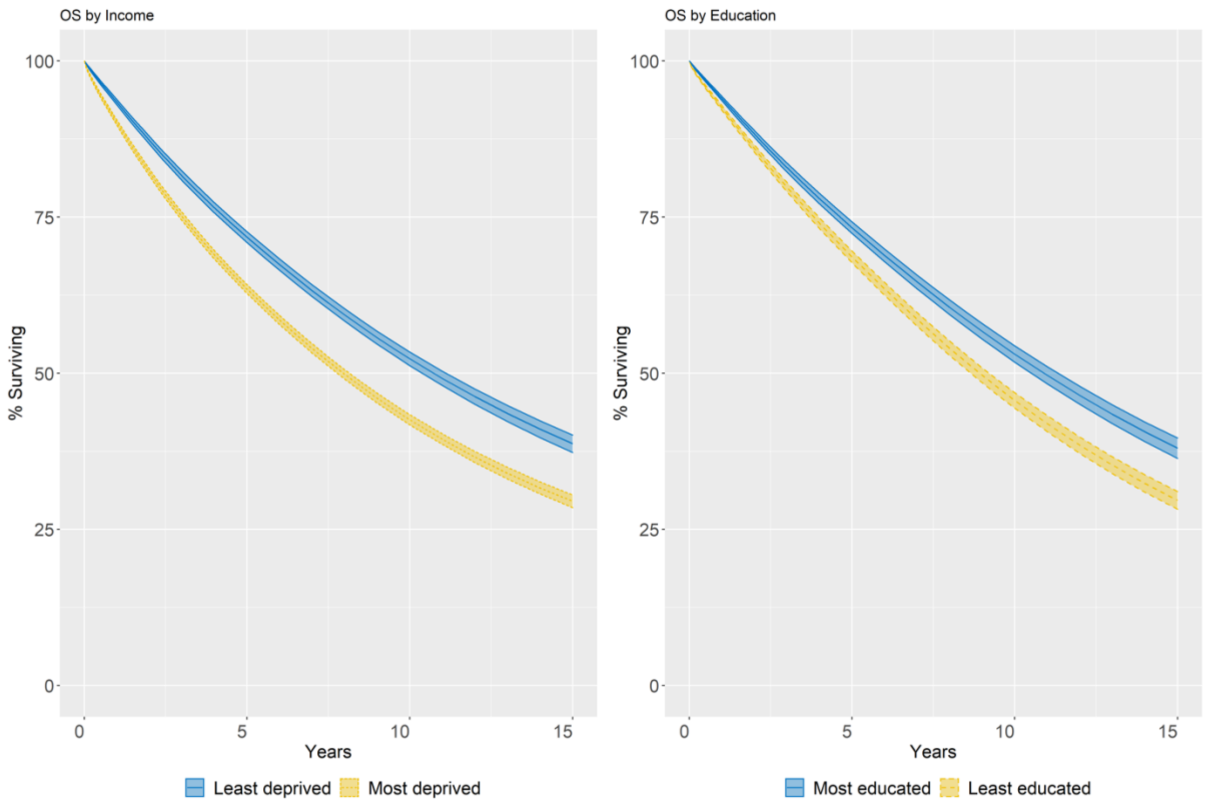

Supplementary Figure 11: Standardised overall survival (OS) for resected colorectal cancer patients with stage I-III disease by deprivation group.

Left panel: Income, Right panel: Education.

Adjusted to the distribution of civil status, year of diagnosis, sex, and age of the most deprived.

Least deprived: Income Q4

Most deprived: Income Q1

Most educated: >12 years of education

Least educated: <9 years of education

Supplementary Table 1: Cut-points for income quartiles in 100 SEK of part of household income averaged to the two years preceding diagnosis

Group	*1st Quartile*	*Median*	*3rd Quartile*
All	1363	1796	2583
Male	1464	1924	2733
Female	1287	1659	2391
<65	1752	2399	3140
>65	1320	1650	2271
Male <65	1747	2431	3173
Male >65	1424	1777	2474
Female <65	1754	2344	3104
Female >65	1249	1513	1928

Supplementary Table 2: All resected colorectal cancer patients diagnosed in Sweden during the years 2008-2021 from CRCBaSe by reason for exclusion and inclusion

Characteristic	Total	Stage IV	Included	Missing pStage	No resection	Stage 0
**Sex**						
Male	47,069 (52.8%)	10,304 (54.4%)	31,298 (52.2%)	948 (50.9%)	4,226 (54.1%)	293 (58.0%)
Female	42,038 (47.2%)	8,621 (45.6%)	28,697 (47.8%)	916 (49.1%)	3,592 (45.9%)	212 (42.0%)
**Age**						
Median (IQR)	73.0 (65.0 - 80.0)	71.0 (63.0 - 78.0)	73.0 (65.0 - 80.0)	74.0 (65.0 - 82.0)	79.0 (70.0 - 86.0)	66.0 (58.0 - 73.0)
**ASA**						
1	9,660 (10.8%)	1,035 (5.5%)	8,280 (13.8%)	213 (11.4%)	26 (0.3%)	106 (21.0%)
2	36,614 (41.1%)	4,320 (22.8%)	31,114 (51.9%)	831 (44.6%)	55 (0.7%)	294 (58.2%)
3	21,340 (23.9%)	2,892 (15.3%)	17,741 (29.6%)	585 (31.4%)	24 (0.3%)	98 (19.4%)
4	2,030 (2.3%)	327 (1.7%)	1,607 (2.7%)	87 (4.7%)	5 (0.1%)	4 (0.8%)
5	38 (0.0%)	3 (0.0%)	31 (0.1%)	4 (0.2%)	0 (0.0%)	0 (0.0%)
Missing	19,425 (21.8%)	10,348 (54.7%)	1,222 (2.0%)	144 (7.7%)	7,708 (98.6%)	3 (0.6%)
**CCI**						
Median (IQR)	0.0 (0.0 - 2.0)	0.0 (0.0 - 2.0)	0.0 (0.0 - 2.0)	0.0 (0.0 - 2.0)	1.0 (0.0 - 2.0)	0.0 (0.0 - 1.0)
Missing	5,306 (6.0%)	1,147 (6.1%)	3,727 (6.2%)	110 (5.9%)	300 (3.8%)	22 (4.4%)
**Location**						
Right	34,109 (38.3%)	7,060 (37.3%)	24,841 (41.4%)	526 (28.2%)	1,672 (21.4%)	10 (2.0%)
Left	25,938 (29.1%)	5,805 (30.7%)	17,195 (28.7%)	427 (22.9%)	2,480 (31.7%)	31 (6.1%)
Rectum	28,656 (32.2%)	5,834 (30.8%)	17,898 (29.8%)	884 (47.4%)	3,576 (45.7%)	464 (91.9%)
Missing	404 (0.5%)	226 (1.2%)	61 (0.1%)	27 (1.4%)	90 (1.2%)	0 (0.0%)
**p/cStage**						
0	549 (0.6%)	0 (0.0%)	0 (0.0%)	0 (0.0%)	44 (0.6%)	505 (100.0%)
1	13,660 (15.3%)	0 (0.0%)	12,897 (21.5%)	0 (0.0%)	763 (9.8%)	0 (0.0%)
2	23,728 (26.6%)	0 (0.0%)	23,612 (39.4%)	0 (0.0%)	116 (1.5%)	0 (0.0%)
3	23,666 (26.6%)	0 (0.0%)	23,486 (39.1%)	0 (0.0%)	180 (2.3%)	0 (0.0%)
4	18,903 (21.2%)	18,903 (99.9%)	0 (0.0%)	0 (0.0%)	0 (0.0%)	0 (0.0%)
Missing	8,601 (9.7%)	22 (0.1%)	0 (0.0%)	1,864 (100.0%)	6,715 (85.9%)	0 (0.0%)
**Income**						
Q1	20,925 (23.5%)	4,641 (24.5%)	13,482 (22.5%)	471 (25.3%)	2,233 (28.6%)	98 (19.4%)
Q2	20,925 (23.5%)	4,357 (23.0%)	13,914 (23.2%)	443 (23.8%)	2,088 (26.7%)	123 (24.4%)
Q3	20,925 (23.5%)	4,322 (22.8%)	14,327 (23.9%)	425 (22.8%)	1,709 (21.9%)	142 (28.1%)
Q4	20,927 (23.5%)	4,430 (23.4%)	14,481 (24.1%)	415 (22.3%)	1,481 (18.9%)	120 (23.8%)
Missing	5,405 (6.1%)	1,175 (6.2%)	3,791 (6.3%)	110 (5.9%)	307 (3.9%)	22 (4.4%)
**Education**						
-9y	22,143 (24.8%)	4,408 (23.3%)	14,609 (24.4%)	503 (27.0%)	2,534 (32.4%)	89 (17.6%)
9y-12y	31,075 (34.9%)	6,737 (35.6%)	20,879 (34.8%)	657 (35.2%)	2,601 (33.3%)	201 (39.8%)
12y-	29,530 (33.1%)	6,412 (33.9%)	20,096 (33.5%)	571 (30.6%)	2,261 (28.9%)	190 (37.6%)
Missing	6,359 (7.1%)	1,368 (7.2%)	4,411 (7.4%)	133 (7.1%)	422 (5.4%)	25 (5.0%)
**Civil**						
Alone	39,268 (44.1%)	8,313 (43.9%)	25,888 (43.2%)	890 (47.7%)	3,994 (51.1%)	183 (36.2%)
Not alone	49,839 (55.9%)	10,612 (56.1%)	34,107 (56.8%)	974 (52.3%)	3,824 (48.9%)	322 (63.8%)

ASA: American Society of Anesthesiologists classification
CCI: Charlson Comorbidity Index
IQR: Interquartile range
Q: Quartile

Supplementary Table 3: Standardised survival estimates for TTR, CSS and OS for the least and most deprived by income (in quartiles) and education (based on the number of years of schooling) at 1, 3, 5 and 10 years for resected colorectal cancer patients diagnosed with stage I-III disease in Sweden during the years 2008-2021 without tumour adjustment

	% (95%CI)	% (95%CI)	% (95%CI)	% (95%CI)
	**1-year TTR**	**3-year TTR**	**5-year TTR**	
**Income**				
Q1	94.4% (94.1-94.7)	85.6% (85.0-86.2)	82.9% (82.2-83.6)	
Q4	94.6% (94.3-95.0)	86.2% (85.5-87.0)	83.6% (82.8-84.4)	
**Education**				
<9 y	94.5% (94.2-94.8)	85.9% (85.2-86.6)	83.2% (82.4-84.0)	
>12y	94.5% (94.2-94.8)	85.9% (85.2-86.6)	83.2% (82.4-84.0)	
	**1-year CSS**	**3-year CSS**	**5-year CSS**	**10-year CSS**
**Income**				
Q1	92.4% (91.9-92.8)	81.0% (80.4-81.7)	73.0% (72.3-73.8)	61.5% (60.5-62.5)
Q4	95.0% (94.7-95.4)	85.7% (85.0-86.4)	78.3% (77.4-79.2)	66.7% (65.5-67.9)
**Education**				
<9 y	93.9% (93.5-94.2)	84.3% (83.6-85.0)	76.9% (76.0-77.8)	65.5% (64.2-66.7)
>12y	95.4% (95.1-95.7)	86.8% (86.2-87.5)	79.8% (78.9-80.7)	68.4% (67.2-69.7)
	**1-year OS**	**3-year OS**	**5-year OS**	**10-year OS**
**Income**				
Q1	90.0% (89.6-90.5)	75.1% (74.4-75.7)	63.5% (62.8-64.2)	42.4% (41.6-43.2)
Q4	92.9% (92.4-93.3)	80.5% (79.7-81.3)	70.4% (69.5-71.2)	50.6% (49.5-51.7)
**Education**				
<9 y	92.4% (92.0-92.8)	79.6% (78.9-80.3)	68.3% (67.4-69.2)	45.2% (44.0-46.3)
>12y	93.6% (93.2-94.0)	82.1% (81.3-82.8)	72.1% (71.2-73.1)	51.6% (50.4-52.9)

Adjusted for civil status, year of diagnosis, sex, age (with restricted cubic splines, 3 knots) and comorbidities using the covariate pattern of the most deprived.

95%CI: 95% Confidence interval
CSS: Cancer-specific survival
OS: Overall Survival
Q: Quartile
TTR: Time to recurrence, recurrence-free proportion reported.

Supplementary Table 4: Standardised survival estimates for TTR, CSS and OS for the least and most deprived by income (in quartiles) and education (based on the number of years of schooling) at 1, 3, 5 and 10 years for resected colorectal cancer patients diagnosed with stage I-III disease in Sweden during the years 2008-2021 without tumour and comorbidity adjustment

	% (95%CI)	% (95%CI)	% (95%CI)	% (95%CI)
	**1-year TTR**	**3-year TTR**	**5-year TTR**	
**Income**				
Q1	94.4% (94.1-94.7)	85.6% (85.0-86.2)	83.0% (82.3-83.7)	
Q4	94.7% (94.4-95.0)	86.4% (85.7-87.1)	83.9% (83.1-84.7)	
**Education**				
<9 y	94.5% (94.2-94.9)	86.0% (85.2-86.7)	83.4% (82.6-84.2)	
>12y	94.6% (94.3-94.9)	86.1% (85.4-86.8)	83.5% (82.7-84.3)	
	**1-year CSS**	**3-year CSS**	**5-year CSS**	**10-year CSS**
**Income**				
Q1	92.4% (92-92.8)	81.3% (80.6-81.9)	73.4% (72.6-74.1)	62.2% (61.2-63.2)
Q4	95.3% (95-95.7)	86.5% (85.8-87.2)	79.5% (78.6-80.4)	68.4% (67.2-69.6)
**Education**				
<9 y	94.0% (93.6-94.3)	84.6% (83.9-85.3)	77.5% (76.6-78.4)	66.5% (65.3-67.7)
>12y	95.7% (95.4-96.0)	87.5% (86.9-88.1)	80.8% (80.0-81.7)	70.0% (68.8-71.2)
	**1-year OS**	**3-year OS**	**5-year OS**	**10-year OS**
**Income**				
Q1	90.1% (89.7-90.6)	75.1% (74.4-75.8)	63.5% (62.8-64.2)	42.6% (41.8-43.4)
Q4	93.4% (92.9-93.8)	81.6% (80.9-82.4)	71.8% (70.9-72.6)	52.3% (51.2-53.4)
**Education**				
<9 y	92.5% (92.1-92.9)	79.8% (79.0-80.5)	68.6% (67.7-69.6)	45.6% (44.4-46.9)
>12y	94.0% (93.6-94.3)	82.9% (82.2-83.6)	73.3% (72.4-74.2)	53.1% (51.8-54.4)

Adjusted for civil status, year of diagnosis, sex, age (with restricted cubic splines, 3 knots) using the covariate pattern of the most deprived.

95%CI: 95% Confidence interval
CSS: Cancer-specific survival
OS: Overall Survival
Q: Quartile
TTR: Time to recurrence, recurrence-free proportion reported.

Supplementary Table 5: Hazard ratios of CSS and OS for least and most deprived by income (in quartiles) and education (based on the number of years of schooling) at 1, 3, 5 and 10 years for resected colorectal cancer patients diagnosed with stage I-III disease in Sweden during the years 2008-2021 without tumour adjustment

	HR (95%CI)	HR (95%CI)	HR (95%CI)	HR (95%CI)
	**1-year CSS**	**3-year CSS**	**5-year CSS**	**10-year CSS**
**Income**				
Q4 vs Q1	0.74 (0.68-0.79)	0.83 (0.79-0.88)	0.89 (0.83-0.95)	0.98 (0.87-1.08)
**Education**				
>12y vs <9 y	0.84 (0.78-0.89)	0.9 (0.86-0.95)	0.93 (0.88-0.99)	0.98 (0.88-1.07)
	**1-year OS**	**3-year OS**	**5-year OS**	**10-year OS**
**Income**				
Q4 vs Q1	0.77 (0.72-0.81)	0.80 (0.77-0.83)	0.81 (0.77-0.84)	0.83 (0.78-0.88)
**Education**				
>12y vs <9 y	0.90 (0.85-0.95)	0.86 (0.83-0.89)	0.83 (0.80-0.86)	0.80 (0.75-0.84)

Adjusted to the distribution of civil status, year of diagnosis, sex, and age and comorbidities of the most deprived
95%CI: 95% Confidence interval
CSS: Cancer-specific survival
HR: Hazard Ratio
OS: Overall Survival
Q: Quartile

Supplementary Table 6: Hazard ratios of CSS and OS for least and most deprived by income (in quartiles) and education (based on the number of years of schooling) at 1, 3, 5 and 10 years for resected colorectal cancer patients diagnosed with stage I-III disease in Sweden during the years 2008-2021 without tumour and comorbidity adjustment

	HR (95%CI)	HR (95%CI)	HR (95%CI)	HR (95%CI)
	**1-year CSS**	**3-year CSS**	**5-year CSS**	**10-year CSS**
**Income**				
Q4 vs Q1	0.70 (0.65-0.75)	0.80 (0.75-0.84)	0.86 (0.8-0.92)	0.95 (0.84-1.06)
**Education**				
>12y vs <9 y	0.81 (0.76-0.86)	0.88 (0.84-0.92)	0.92 (0.86-0.97)	0.97 (0.88-1.06)
	**1-year OS**	**3-year OS**	**5-year OS**	**10-year OS**
**Income**				
Q4 vs Q1	0.72 (0.67-0.76)	0.76 (0.72-0.79)	0.77 (0.74-0.81)	0.81 (0.76-0.86)
**Education**				
>12y vs <9 y	0.86 (0.81-0.90)	0.83 (0.80-0.87)	0.81 (0.78-0.84)	0.78 (0.74-0.83)

Adjusted to the distribution of civil status, year of diagnosis, sex, and age of the most deprived
95%CI: 95% Confidence interval
CSS: Cancer-specific survival
HR: Hazard Ratio
OS: Overall Survival
Q: Quartile
